# *Nannochloropsis oceanica* Lipid Extract Moderates UVB-Irradiated Psoriatic Keratinocytes: Impact on Protein Expression and Protein Adducts

**DOI:** 10.3390/antiox13101236

**Published:** 2024-10-14

**Authors:** Adam Wroński, Agnieszka Gęgotek, Tiago Conde, Maria Rosário Domingues, Pedro Domingues, Elżbieta Skrzydlewska

**Affiliations:** 1 Dermatological Specialized Center “DERMAL” NZOZ in Bialystok, Nowy Swiat 17/5, 15-453 Bialystok, Poland; 2 Department of Analytical Chemistry, Medical University of Bialystok, Mickiewicza 2d, 15-222 Bialystok, Poland; elzbieta.skrzydlewska@umb.edu.pl; 3 Mass Spectrometry Centre, LAQV-REQUIMTE, Department of Chemistry, University of Aveiro, Santiago University Campus, 3810-193 Aveiro, Portugal; tiagoalexandreconde@ua.pt (T.C.); mrd@ua.pt (M.R.D.); p.domingues@ua.pt (P.D.); 4 Centre for Environmental and Marine Studies, Department of Chemistry, University of Aveiro, Santiago University Campus, 3810-193 Aveiro, Portugal

**Keywords:** keratinocytes, psoriasis, UVB radiation, protein adducts, lipid extract, microalgae *Nannochloropsis oceanica*

## Abstract

Psoriasis is characterized by excessive exfoliation of the epidermal layer due to enhanced pro-inflammatory signaling and hyperproliferation of keratinocytes, further modulated by UV-based anti-psoriatic treatments. Consequently, this study aimed to evaluate the impact of a lipid extract derived from the microalgae *Nannochloropsis oceanica* on the proteomic alterations induced by lipid derivatives in non-irradiated and UVB-irradiated keratinocytes from psoriatic skin lesions compared to keratinocytes from healthy individuals. The findings revealed that the microalgae extract diminished the viability of psoriatic keratinocytes without affecting the viability of these cells following UVB exposure. Notably, the microalgae extract led to an increased level of 4-HNE-protein adducts in non-irradiated cells and a reduction in 4-hydroxynonenal (4-HNE)-protein and 15-deoxy-12,14-prostaglandin J2 (15d-PGJ2)-protein adducts in UVB-exposed keratinocytes from psoriasis patients. In healthy skin cells, the extract decreased the UV-induced elevation of 4-HNE-protein and 15d-PGJ2-protein adducts. The antioxidant/anti-inflammatory attributes of the lipid extract from the *Nannochloropsis oceanica* suggest its potential as a protective agent for keratinocytes in healthy skin against UVB radiation’s detrimental effects. Moreover, it could offer therapeutic benefits to skin cells afflicted with psoriatic lesions by mitigating their proliferation and inflammatory responses during UV radiation treatment.

## 1. Introduction

The most external organ of the human body—skin, is involved in the basic protection of the body against harmful external factors, but also ensures communication with the surrounding environment. Therefore, its condition is so important for the body to properly function, that any disorders in its structure pose a serious health risk to the entire organism [1]. One of the most common non-cancerous skin diseases is psoriasis, which, according to the WHO data, is diagnosed in up to 4% of the human population [2]. Skin psoriatic lesions are characterized by red, scaly plaques, causing significant physical/psychological burdens for affected patients [3]. The pathogenesis of psoriasis involves complex interactions between genetic, immunological, and environmental factors, leading to dysregulated immune responses and excessive proliferation of keratinocytes [4]. So far, the changes in the metabolism of keratinocytes from the skin of psoriatic patients have been examined in great detail, both at the lipid and protein levels [5,6]. Moreover, the changes occurring in the metabolism of cells of the immune system [5], as well as their reflection in the plasma of psoriatic patients [7] are also partially known. In general, the development of psoriasis is associated with increased pro-inflammatory signaling, disruption in the antioxidant system, impaired autophagy, overproduction of growth factors and accelerated apoptosis [8,9]. All these changes are linked to intra/extracellular signalization [10], in particular to protein modification by-products of lipid metabolism [11]. Moreover, the mentioned protein modification by-products of lipid metabolism occur not only in the epidermis, but also in dermal fibroblasts, and are spread throughout the body through immune system cells and plasma. This leads to many comorbidities associated with psoriasis, including psoriatic arthritis, diabetes, dyslipidaemia, obesity, hypertension, metabolic syndrome, non-alcoholic fatty liver disease, inflammatory bowel disease, and kidney disease [12]. Additionally, psoriatic patients have an increased risk of hospitalization due to infection and their mortality from other diseases is generally approximately 2% higher [13].

The traditional treatment of psoriatic skin lesions involves their exposure to UV radiation (UVA or UVB) [14]. Regardless of the therapy used, skin cells, including those affected by psoriasis, are also regularly exposed to UV radiation present in sunlight, which further promotes the development of oxidative stress and consequently the oxidation of intracellular molecules, including proteins and lipid peroxidation [15]. This process promotes the generation of pro-inflammatory signaling molecules, including small, molecular reactive aldehydes and neuro-isoprostanes [16]. Moreover, UV radiation increases the activity of enzymes involved in lipid metabolism, including phospholipases, as well as the transformation of free polyunsaturated fatty acids (PUFAs) into eicosanoids by cyclooxygenases, lipoxygenases, cytochrome P450, and terminal prostanoid synthases [17,18]. Eicosanoids ultimately formed in skin cells may mediate inflammatory events developed in response to environmental factors, such as exposure to UV and inflammatory and allergic disorders, including psoriasis and atopic dermatitis [17]. Therefore, there is still a need for effective compounds that can prevent the development of skin psoriasis and/or have a regenerative effect and reverse the changes occurring in the skin and throughout the body. Due to the above-mentioned range of metabolic changes associated with psoriasis, natural antioxidants are constantly being sought after as medicinal compounds that prevent protein oxidation and inhibit pro-inflammatory signaling resulting from changes in the level of lipid metabolites.

Research in recent years indicates that the most effective multi-directional metabolic action is not exerted by isolated protective compounds but by their mixtures. These compounds can be found, for example, in the composition of lipid extracts of microalgae, whose potential to protect skin cells has already been proven [19]. Microalgae extracts are natural products rich in bioactive compounds, such as phospholipids, PUFAs, polysaccharides, and vitamins, which are increasingly proposed for use in the protection of human health [20,21]. Its activity has been mainly linked to their antioxidant properties, but also to their capacity to regulate lipid metabolism, and to inhibit the production of pro-inflammatory cytokines, including tumor necrosis factor alfa (TNFα), interleukins (IL-1β and IL-6), as well as alter nitric oxide synthase (NOS) activity, leading to the suppression of inflammation [19]. Additionally, they can act as anti-proliferative factors, which has been documented in the case of melanomas, but also lung, colorectal, breast, and prostate cancer cells [22,23]. However, among different microalgae species, the resulting extracts differ in composition and levels of bioactive compounds and may have different therapeutic properties.

One of the most promising extracts in the context of skin protection in various stress conditions is the lipid extract obtained from the marine algae *Nannochloropsis oceanica.* So far, studies have demonstrated that the lipid extracts of this microalgae have antioxidant, anti-inflammatory, and restorative effects in the case of skin fibroblasts and keratinocytes exposed to UVB radiation [24,25]. Consequently, this investigation sought to examine the impact of a lipid extract from the *Nannochloropsis oceanica* on the proteome of keratinocytes. It aimed to assess protein modifications by lipid derivatives in both non-irradiated and UVB-irradiated cells derived from psoriatic skin lesions and to compare these findings with the proteome of similarly treated keratinocytes obtained from healthy donors.

## 2. Materials and Methods

### 2.1. Material Collection

Skin tissues, from which keratinocytes were derived, were collected from 5 untreated patients with a diagnosis of psoriasis vulgaris (3 men and 3 women; age range 23–58 years, mean age 44) and 5 healthy volunteers (sex-matched individuals forming a control group; age range 21–64 years, mean age 45). Patients from whom samples were collected were diagnosed with plaque psoriasis for at least six months before the study and had at least 10% of their total body surface affected. None of the patients or healthy donors received topical, injectable, or oral medications for 4 weeks before the study. None of the study participants were smokers, excessive consumers of alcohol, or had a history indicating the presence of other disorders. The study was conducted following the Declaration of Helsinki, and the protocol was approved by the Local Bioethics Committee of the Medical University of Bialystok (Poland), No. APK.002.454.2023. Written informed consent was obtained from all participants.

### 2.2. Microalgae Lipid Extracts

The microalgae *Nannochloropsis oceanica* were cultivated in Guillard’s F2 culture medium adapted to the local water as described previously [25]. The microalgae biomass was spray-dried and supplied by Allmicroalgae, Natural Products S.A. located in Rua 25 de Abril s/n 2445-413 Pataias, Portugal.

The microalgae biomass was mixed with dichloromethane/methanol (2:1, *v*/*v*) and centrifuged (670× *g*) for 10 min to obtain supernatant, which was repeated three times to optimize extraction. To remove any non-lipid fractions, the extracts were vortexed with Mili-Q water and following centrifugation (670× *g* for 10 min), the organic fraction was collected. The lipid profile of the obtained *Nannochloropsis oceanica* extract was characterized by hydrophilic interaction liquid chromatography coupled with high-resolution mass spectrometry (HILIC-MS) and tandem MS (MS/MS) using a Q-Exactive hybrid quadrupole Orbitrap mass spectrometer (Thermo Fisher Scientific, Bremen, Germany), as reported previously (Supplementary Table S1) [26].

### 2.3. Cell Culture and Treatment

Skin biopsies were washed in PBS with penicillin (50 U/mL) and streptomycin (50 μg/mL), and incubated overnight at 4 °C in dispase (1 mg/mL) to separate the epidermis from the dermis. Next, the obtained epidermis was digested for 20 min at 37° using trypsin/EDTA. Isolated keratinocytes were resuspended and cultured in a growing culture medium: Keratinocyte Serum-Free Medium (Gibco, Grand Island, NY, USA) containing foetal bovine serum (10%), epidermal growth factor EGF 1-53 (5 µg/L), penicillin (50 U/mL), and streptomycin (50 μg/mL). When cells reached 80% confluence, they were exposed to UV radiation in cold PBS (4 °C). Bio-Link Crosslinker BLX 312 (Vilber Lourmat, Eberhardzell, Germany) with the 6 lamps assembly at 6 W each was used to irradiate cells. The exposure dose was set to 60 mJ/cm^2^ and was chosen corresponding to 70% cell viability measured by the MTT assay [27].

To examine the effect of the lipid extract from the *Nannochloropsis oceanica*, following irradiation, cells were incubated for 24 h in a medium supplemented with the extract at a concentration of 3 µg/mL, primarily dissolved in DMSO (dimethyl sulfoxide), which in the final solution was 0.1%. The concentration of the extract was selected based on previous studies on healthy keratinocytes (human immortalized keratinocytes CDD 1102 KERTr (CRL2310, American Type Culture Collection, Manassas, VA, USA)). It was found that, at this concentration, the algae extract did not induce changes in the viability of non-irradiated cells, and at the same time significantly protected keratinocytes against cell death induced by UVB radiation [25]. Control cells (non-irradiated or UVA-irradiated) were cultured in parallel in a medium containing 0.1% DMSO.

After incubation, keratinocytes were collected by scraping into cold PBS and subjected to lysis by sonification. The total protein content in the cell lysate was measured using a Bradford reagent.

### 2.4. In-Gel Protein Separation

To compare the distribution and quantity of proteins in cell lysates, their preliminary separation was carried out using SDS-PAGE. Gels were stained overnight with Coomassie Brilliant Blue R-250 and then rinsed in the methanol/acetic acid/water (3:1:6).

### 2.5. In-Solution Protein Digestion and Peptide Analysis

Samples containing 50 µg proteins were denatured by mixing with 8 M urea, reduced with 10 mM 1,4-dithiothreitol (DTT) and alkylated with 50 mM iodoacetamide (IAA). Before overnight (37 °C) digestion with trypsin (Promega, Madison, WI, USA), samples were fourfold diluted with ammonium bicarbonate buffer (AMBIC, 25 mM). To stop the reaction, 0.1% formic acid (FA) was added. The obtained peptide mixture, after drying, was dissolved in 5% acetonitrile with 0.1% FA. This mixture was injected and separated on an analytical column (50 mm × 75 µm PepMap RSLC capillary C18 column) connected with the high-performance liquid chromatography system Ultimate 3000 (Dionex, Idstein, Germany). Following separation, peptide masses were analyzed using a Q Exactive HF mass spectrometer (Thermo Fisher Scientific, Bremen, Germany) operated by Xcalibur 4.1 software (supplied by the same supplier). Analysis parameters in detail have been described previously [7].

### 2.6. Protein Identification and Label-Free Quantification

Raw data were analyzed using the freeware software MaxQuant v2.4.2. Input data were searched against the UniProtKB-SwissProt database (taxonomy: Homo sapiens, release 2023-09). The default parameters were used for protein identification. Additional dynamic modification of cysteine/lysine/histidine by 4-hydroxynonenal (4-HNE) or 15-deoxy-12,14-prostaglandin J2 (15d-PGJ2) was set, according to the Unimod Protein Modifications for Mass Spectrometry Database [28]. Protein quantification was performed based on the peak area analysis.

### 2.7. Statistical Analysis

Each cell variant was repeated in 5 independent biological replicates. The results from individual protein label-free quantifications were log transformed, auto-scaled (mean centered and divided by the standard deviation of each variable), and normalized by the sum of the protein intensities obtained for each sample using open-source software MetaboAnalyst 6.0 (http://www.metaboanalyst.ca, access date: 5 March 2024) [29]. Biostatistical analysis, including ANOVA (one-way univariate analysis of variance with a false discovery rate (FDR) < 5%), but also data visualization based on heatmap and dendrogram creation were also prepared using MetaboAnalyst 6.0. PANTHER 18.0 database (Protein ANalysis THrough Evolutionary Relationships Classification System) was used for protein function determination.

## 3. Results

**Figure 1:**
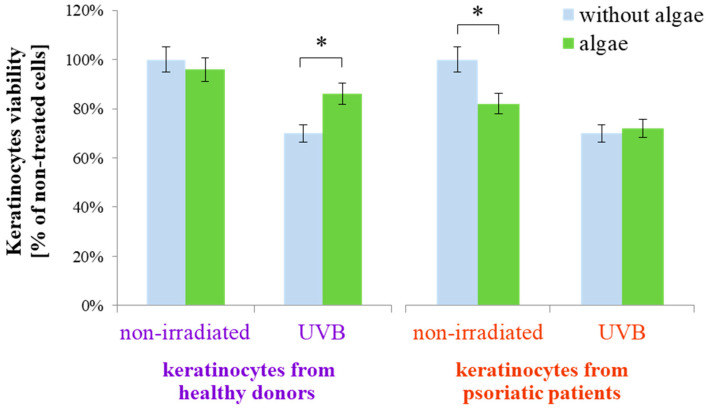
Viability of keratinocytes isolated from the skin of healthy donors and psoriatic patients after UVB irradiation (60 mJ/cm^2^) and 24 h incubation with a lipid extract of the microalgae *Nannochloropsis oceanica* (3 ng/mL). Mean values ± SD are presented with statistically significant differences (cells treated with microalgae extract vs. equally irradiated/non-irradiated keratinocytes from healthy donors/psoriatic patients, * *p* < 0.05).

The results obtained indicated that the lipid extract of the microalgae Nannochloropsis oceanica differently affects the metabolism of keratinocytes isolated from healthy people and psoriatic patients in both cases, without irradiation and after exposure to UVB radiation. This was observed even at the experimental level of keratinocyte viability (Figure 1). Keratinocytes from healthy donors, when treated with the microalgae extract, exhibited no change in viability compared to untreated cells. However, the viability reduced by UVB irradiation was partially restored with the application of the extract. In contrast, for keratinocytes from psoriatic patients not subjected to UVB radiation, the microalgae lipid extract decreased the viability of non-irradiated cells but did not significantly affect the viability of keratinocytes exposed to UVB radiation from psoriatic patients.

**Figure 2:**
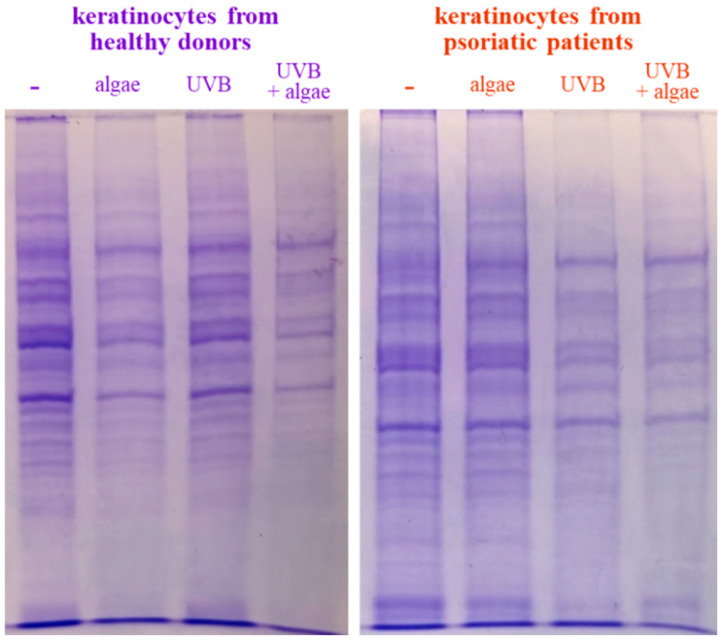
SDS-PAGE analysis of proteins from keratinocytes isolated from the skin of healthy donors and psoriatic patients after UVB irradiation (60 mJ/cm^2^) and 24 h incubation with a lipid extract of the microalgae *Nannochloropsis oceanica* (3 ng/mL).

**Figure 3:**
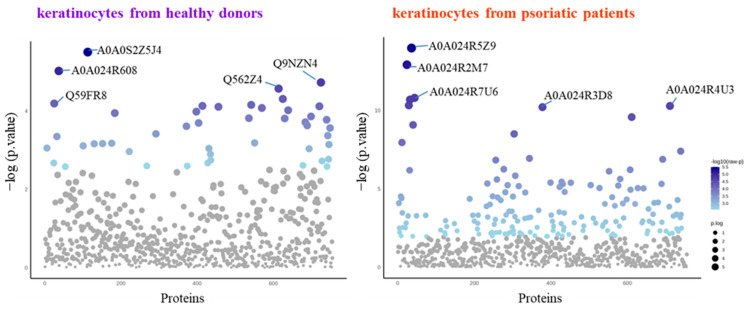
Results of one-way univariate analysis of variance (ANOVA) of the proteins in keratinocytes isolated from the skin of healthy donors and psoriatic patients after UVB irradiation (60 mJ/cm^2^) and 24 h incubation with a lipid extract of the microalgae *Nannochloropsis oceanica* (3 ng/mL). The IDs of the top 5 most significantly different proteins are marked on the plots: A0A024R2M7, oxidative-stress responsive 1; A0A024R3D8, pyruvate dehydrogenase; A0A024R4U3, tubulin tyrosine ligase; A0A024R5Z9, pyruvate kinase; A0A024R608, ribosomal protein; A0A024R7U6, DNA replication licensing factor; A0A0S2Z5J4, adaptor-related protein complex 3; Q562Z4, actin-like protein; Q59FR8, galectin; Q9NZN4, EH domain-containing protein 2. The IDs and *p*-values for the rest of the statistically significant proteins are presented in Supplementary Table S3.

**Figure 4:**
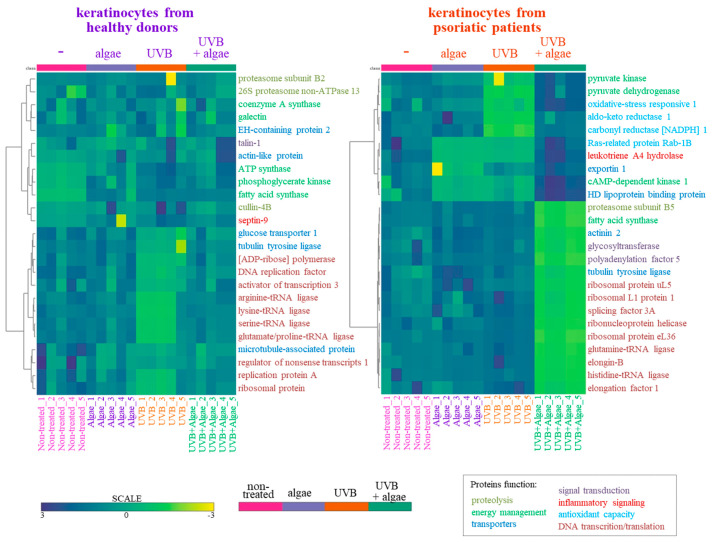
Heatmap and clustering of the top 25 changing proteins in keratinocytes isolated from the skin of healthy donors and psoriatic patients after irradiation with UVB (60 mJ/cm^2^) and 24 h incubation with a lipid extract of the microalgae *Nannochloropsis oceanica* (3 ng/mL). The functions of the proteins marked with colors described in the bottom right corner are assigned based on the Protein ANalysis THrough Evolutionary Relationships Classification System (PANTHER 18.0).

The SDS-PAGE of protein samples showed significant differences in band distribution only between keratinocytes isolated from healthy donors and psoriatic patients, which was discussed earlier [5], and there was no significant differences between cells non-treated and treated with the algae extract (Figure 2). Further analyses using mass spectrometry (MS) based proteomics identified significant changes in the proteome of the cells examined. This approach allowed us to identify and semi-quantify 755 proteins found in each sample (Supplementary Table S2) The ANOVA analysis revealed statistically significant alterations in the expression of 47 proteins in the group of keratinocytes from healthy donors treated with UVB and the lipid extract. Similarly, in keratinocytes from psoriatic patients receiving the same treatment, the expression of 168 proteins was significantly changed (Figure 3). The most notable changes in protein expression included the following: adaptor-related protein complex 3 (A0A0S2Z5J4), ribosomal protein (A0A024R608), galectin (Q59FR8), actin-like protein (Q562Z4), EH domain-containing protein 2 (Q9NZN4), pyruvate kinase (A0A024R5Z9), oxidative-stress responsive 1 (A0A024R2M7), DNA replication licensing factor (A0A024R7U6), pyruvate dehydrogenase (A0A024R3D8), and tubulin tyrosine ligase (A0A024R4U3). The top 25 most significantly modified proteins in each group were utilized to build the heatmaps (Figure 4). In keratinocytes from both healthy donors and psoriasis patients, these proteins were clustered into three main groups, with the larger clusters predominantly comprising proteins involved in DNA transcription and translation.

**Figure 5:**
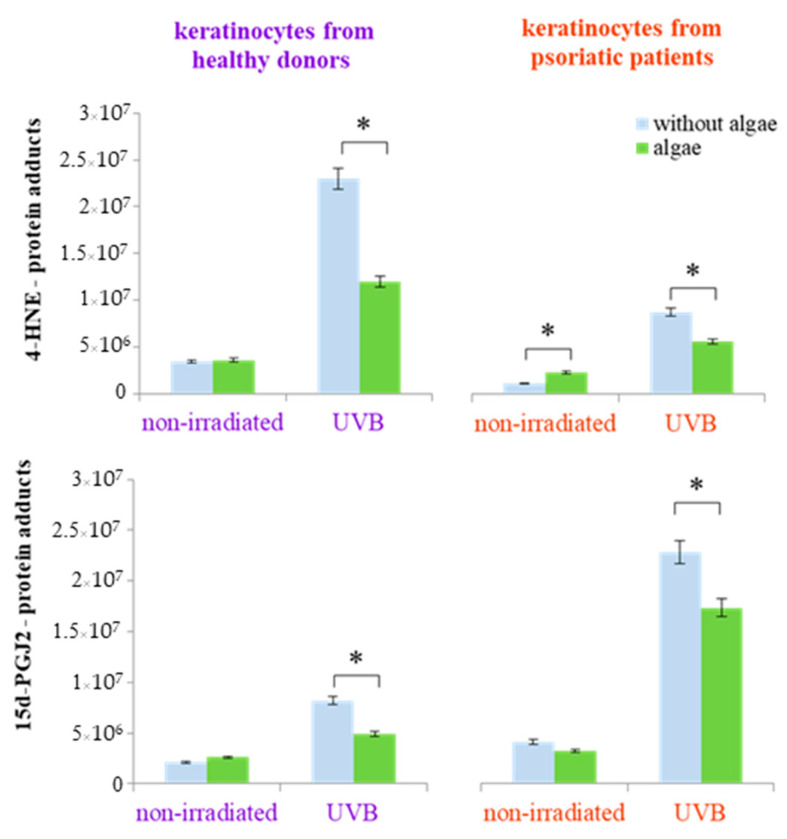
The total level of protein modifications by lipid/PUFAs metabolism products (4-hydroxynonenal (4-HNE) and 15-deoxy-12,14-prostaglandin J2 (15d-PGJ2)) in keratinocytes isolated from the skin of healthy donors and psoriatic patients after irradiation with UVB (60 mJ/cm^2^) and 24 h incubation with a lipid extract of microalgae *Nannochloropsis oceanica* (algae, 3 ng/mL). Mean values ± SD are shown annotated with statistically significant differences (cells treated with microalgae extract vs. irradiated/non-irradiated keratinocytes from healthy donors/psoriatic patients, * *p* < 0.05).

The lipid extract from the Nannochloropsis oceanica not only affected the expression of individual proteins but also influenced protein structure, including modifications through the formation of protein adducts with lipid metabolism products (4-HNE and 15d-PGJ2-protein adducts). This effect was not observed in keratinocytes from healthy donors that were not irradiated. However, following UVB radiation exposure, the microalgae extract significantly reduced the elevated levels of 4-HNE-protein adducts (by approximately 50%) and 15d-PGJ2-protein adducts (by approximately 30%) (Figure 5). In contrast, keratinocytes from psoriatic patients showed an increase in the level of 4-HNE-protein adducts in non-irradiated cells and a reduction of approximately 25% in the levels of both 4-HNE-protein and 15d-PGJ2-protein adducts in UVB-exposed cells (Figure 5). In addition to the alterations in the levels of protein adducts with lipid metabolism products, UVB radiation also induced changes in the various types of proteins depending on whether the cells originated from healthy donors or psoriatic patients. These changes were, to varying degrees, reversible through the treatment of the cells with the algae extract (Table 1 and Table 2).

## 4. Discussion

The skin barrier serves as the primary defense against external factors. Consequently, natural substances or compounds that offer protective and regenerative benefits to skin cells subjected to different physicochemical factors are in great demand [30]. Marine microalgae harbor unique compounds enabling cellular adaptation to different natural conditions [31]. Thus, the potential of these extracts, particularly lipid extracts, in skin protection and the therapeutic processes (including UV radiation) for skin diseases, such as psoriasis [32], warrants thorough investigation. Furthermore, lipid extracts exhibit a superior ability to scavenge free radicals compared to non-polar extracts in keratinocytes after UVB radiation exposure [31]. The systemic effects of various algae species (administered via oral supplementation) were recently shown to result in a significant decrease in the levels of inflammatory cytokines in the blood of psoriasis patients [33]. Additionally, local treatment of psoriasis with algae compounds has also shown promising results. Carotenoids from *Alkalinema* sp., *Cyanobium* sp., *Nodosilinea* sp. or *Cuspidothrix* sp. applied to psoriatic skin showed anti-inflammatory and antiproliferative effects [34]. Simultaneously, in vitro treatment of skin cells with extracts of *Micractinium* sp., *Chlamydomonas* sp., and *Chlorococcum* sp. algae have shown that the mechanism of their antioxidant and anti-inflammatory action is mainly due to the inhibition of the biosynthesis and release of pro-inflammatory cytokines [31]. Therefore, the primary objective of this study was to investigate the impact of lipid extract from the *Nannochloropsis oceanica* microalgae on the proteomic profile of keratinocytes from psoriatic patients, compared to cells from healthy individuals. Special attention was given to the modification of proteins by the products of lipid metabolism (4-HNE and 15d-PGJ2).

The results from the proteomic analysis obtained in this study (visualized in the heatmap, Figure 4), clearly indicated that the lipid extract induced significant changes in the proteome of untreated keratinocytes isolated from healthy subjects, mainly through the upregulation of ATP synthase, phosphoglycerate kinase, fatty acid synthase, cullin, and septin-9 expression. The first three of these proteins are involved in cellular energy metabolism [35,36,37], while septin-9 is responsible for intracellular signal transduction, including the regulation of cell division, as well as the expression of cullin and further protein polyubiquitination [38]. It can therefore be suggested that the metabolism of healthy keratinocytes might be enhanced, leading to increased energy levels within the cells, facilitating more frequent cell divisions along with the simultaneous degradation of redundant labelled molecules [39]. A contrasting scenario is observed in UVB-irradiated keratinocytes, where changes in the proteomic profile have previously been discussed [40]. Treatment with the lipid extract led to an increase in the UVB-diminished expression of proteins, primarily those involved in DNA transcription and translation, effectively restoring their levels to that of control cells. This effect was particularly evident in the extensive group of diverse tRNA ligases, indicating that the response of these cells to irradiation involves accelerating the biosynthesis of new, unoxidized proteins, including enzymes critical for repair and cellular protection [41].

The lipid extract exhibited a distinct effect on the proteome of keratinocytes isolated from psoriatic patients. Significant differences were noted in the expression of proteins such as Ras-related protein Rab-1B, leukotriene A4 hydrolase, exportin 1, cAMP-dependent kinase 1, and high-density lipoprotein binding protein, which were reduced following treatment with the extract. Given the elevated levels of leukotriene (LTB4) in psoriatic skin, the presence of a potential leukotriene A4 hydrolase inhibitor in the *Nannochloropsis oceanica* extract could represent a promising compound in the treatment of psoriasis [42]. The algae extract significantly decreased the expression of proteins primarily involved in DNA transcription and translation within these cells, and inhibited the biosynthesis of new proteins, thereby further slowing down keratinocyte proliferation. Concurrently, the lipid extract also protected against the UVB-induced reduction in a wide group of proteins such as Aldo-keto reductase 1, oxidative-stress responsive 1, and carbonyl reductase NADPH 1, which play crucial roles in antioxidant protection of the cells [43], as well as pro-inflammatory Ras-related protein Rab-1B and leukotriene A4 hydrolase [44]. Consequently, UVB-irradiated keratinocytes from psoriatic patients, in addition to inhibiting proliferation, have increased antioxidant protection against premature apoptosis. This effect is supported by the activity of pyruvate kinase/pyruvate dehydrogenase, whose expression, stimulated by the algae extract, ensures efficient energy management in cells irradiated by UVB.

The lipid extract from the microalgae also impacted the proteome of the keratinocytes by influencing the protein structure, particularly through the formation of adducts with products of lipid metabolism, including 4-HNE and 15d-PGJ2. Cell treatment with lipid extract, especially following UVB irradiation, significantly reduces the level of 4-HNE and 15d-PGJ2—protein adducts in keratinocytes isolated from healthy donors, which is accompanied with the cells’ protection against decreased viability. This observation is closely related to the strong antioxidant and anti-inflammatory properties of the microalgae extract mentioned above [24,25,45]. Simultaneously, the same protective effect is not observed in keratinocytes from psoriatic patients, whose overexpression is driven by pro-inflammatory cytokines, partially silenced by components of the lipid extract [31].

As previously described, 4-HNE-protein adducts play an important role in the development of psoriasis, by activating the antioxidant system and inducting an inflammatory response [11]. However, the results obtained in this study reveal that the microalgae lipid extract effectively prevents the generation of 4-HNE-protein adducts in keratinocytes following UVB irradiation, particularly in cells isolated from healthy donors. This effect is notably observed in proteins such as cullin-3, dipeptidyl peptidase 3, ubiquitin carboxyl-terminal hydrolase 7, AP-2 complex, serpin A12, IκB kinase, and G1/S-specific cyclin-D2. The first four of these proteins are known to indirectly affect the activity of the Nrf2 transcription factor, either by marking for proteolysis or by forming complexes with DNA [46]. While the impact of 4-HNE adduct formation on the activity of these proteins is unclear, other data suggest that the algae extract promotion of Nrf2 activation in UVB-irradiated keratinocytes was reduced [25]. Additionally, the other three proteins directly involved in mediating pro-inflammatory signaling (serpin A12, IκB kinase, and G1/S-specific cyclin-D2) experienced reduced 4-HNE-protein adduct formation due to the lipid extract in UVB-irradiated keratinocytes from healthy donors. This could be the cause of the observed inhibition of pro-inflammatory signaling through the TNFα/NFκB pathway shutdown [25].

Interestingly, the level of changes observed in UVB-irradiated keratinocytes from psoriatic lesions was significantly lower and is related to the prevention of the formation of 4-HNE adducts with serpin A12 and IκB kinase. This mechanism reduces pro-inflammatory signaling without affecting the Nrf2-dependent pathway, unlike in cells from healthy donors. Furthermore, in keratinocytes from psoriatic patients irradiated with UVB, the microalgae extract induced the formation of 4-HNE-protein adducts with adenosine kinase and MAPK10 (JNK3). The consequence of the formation of 4-HNE adducts with adenosine kinase remains unclear. However, for JNK3, it is well described that 4-HNE activates it, leading to the phosphorylation and activation of c-Jun, as well as caspase-dependent apoptosis [47,48]. A similar effect observed in keratinocytes from healthy donors after UVB irradiation likely contributes to decreased cell viability after UVB exposure.

Another type of protein modification significantly altered under experimental conditions is the formation of 15d-PGJ2-protein adducts. 15d-PGJ2, the most recently identified prostaglandin known for its anti-inflammatory properties, exerts its biological activity through the formation of specific protein adducts [49,50]. This study reveals that the lipid extract from microalgae does not significantly influence the level of 15d-PGJ2-protein adducts in non-irradiated keratinocytes from both healthy donors and psoriatic patients. However, following UVB exposure, the extract markedly reduces this modification. In keratinocytes from healthy donors, UVB exposure induces the formation of 15d-PGJ2 adducts with proteins such as those involved in the cell division cycle, apoptosis regulator protein 1, apoptogenic protein 1, signal transducer and activator of transcription 3 (STAT3), PPARγ, and G-protein-coupled receptor 55, a process inhibited by the microalgae lipid extract. Notably, in keratinocytes from psoriatic patients, the proteins related to the cell division cycle and apoptosis regulator, as well as apoptogenic protein 1, were unaffected by the algae extract and did not alter the viability of keratinocytes post-UVB exposure.

Furthermore, the microalgae extract prevented the UVB-induced formation of 15d-PGJ2 adducts with STAT3, PPARγ, and G-protein-coupled receptor 55, impacting cell growth and death processes in keratinocytes from both healthy donors and psoriatic patients. The formation of 15d-PGJ2-PPARγ and 15d-PGJ2-G-protein-coupled receptor 55 adducts activates PPARγ and G-protein-coupled receptor 55, leading to the inhibition of cell growth [51,52]. Consequently, UVB-irradiated cells exhibited inhibited growth and blocked apoptosis, whereas the lipid extract from microalgae prevented these metabolic alterations in skin cells, irrespective of their origin from healthy or psoriatic skin.

## 5. Conclusions

Summarizing, the antioxidant and anti-inflammatory properties of the lipid extract from the *Nannochloropsis oceanica* suggest that it is a promising protective agent for keratinocytes in healthy skin exposed to the detrimental effects of UVB radiation. Moreover, this extract may also benefit skin cells affected by psoriatic lesions, reducing cell accumulation in the exfoliated epidermis and alleviating inflammatory processes during UV radiation therapy. These effects stem from the microalgae lipid extract’s impact not only on protein expression but also on the modification of protein structure, thereby influencing intracellular signaling. A significant advantage of this extract is its differentiated effect on the viability of keratinocytes isolated from healthy individuals versus those from psoriatic patients, where it specifically inhibited the proliferation of psoriatic skin cells.

## Figures and Tables

**Table 1:** The list of proteins modified by lipid peroxidation product (4-hydroxynonenal (4-HNE)) in each keratinocyte experimental group isolated from the skin of healthy donors (marked as a purple X) and psoriatic patients (marked as red X) after irradiation with UVB (60 mJ/cm^2^) and 24 h incubation with a lipid extract of microalgae *Nannochloropsis oceanica* (algae, 3 ng/mL). X indicates that adducts were identified in each sample group without comparing their quantity between groups.

4-HNE-Modified Protein	Keratinocytes from Healthy Donors and Psoriatic Patients			
	Non-Irradiated		UVB	
	Without Algae	Algae	Without Algae	Algae
serine/threonine-protein phosphatase 2A	x x	x x		
interferon-induced protein kinase	x x	x x		
histone acetyltransferase	x x	x x		
NADH-cytochrome reductase	x x	x x	x	
p62	x	x x		
protein kinase C		x	x x	x x
cullin-3		x	x x	x
dipeptidyl peptidase 3		x	x x	x
serpin A12			x x	
ubiquitin carboxyl-terminal hydrolase 7			x	x
IκB kinase			x x	
adenosine kinase			x	x x
mitogen-activated protein kinase 10			x	x x
ribosomal protein S6 kinase			x x	x x
AP-2 complex			x	
G1/S-specific cyclin-D2			x x	

**Table 2:** The list of proteins modified by-product of PUFAs metabolism (15-deoxy-12,14-prostaglandin J2 (15d-PGJ2)) in each keratinocytes experimental group isolated from the skin of healthy donors (marked as purple X) and psoriatic patients (marked as red X) after irradiation with UVB (60 mJ/cm^2^) and 24 h incubation with a lipid extract of microalgae *Nannochloropsis oceanica* (algae, 3 ng/mL). X indicates that adducts were identified in each sample group without comparing their quantity between groups.

15d-PGJ2-Modified Protein	Keratinocytes from Healthy Donors and Psoriatic Patients			
	Non-Irradiated		UVB	
	Without Algae	Algae	Without Algae	Algae
p53	x x	x x	x x	x x
apoptosis inhibitor 5	x x	x x	x x	x x
cell division cycle and apoptosis regulator protein 1	x x	x x	x x	x
apoptogenic protein 1	x x	x x	x x	x
IκB kinase	x		x	x x
collapsin response mediator protein 1			x x	x x
signal transducer and activator of transcription 3			x x	
PPARγ			x x	
G-protein-coupled receptor 55			x x	
prostanoid FP receptor			x	x x
integrin A7			x	x

## Data Availability

The authors confirm that the data supporting the findings of this study are available within the article and its Supplementary Materials.

## Supplementary Materials

The following supporting information can be downloaded at https://www.mdpi.com/article/10.3390/antiox13101236/s1. Supplementary Table S1: The lipid profile of the obtained *Nannochloropsis oceanica* extract characterized by hydrophilic interaction liquid chromatography coupled with high-resolution mass spectrometry (HILIC-MS) and tandem MS (MS/MS) using a Q-Exactive hybrid quadrupole Orbitrap mass spectrometer (Thermo Fisher Scientific, Bremen, Germany). Supplementary Table S2: The IDs of the proteins that were identified and semi-quantified in keratinocytes isolated from the skin of healthy donors and psoriatic patients after irradiation with UVB (60 mJ/cm^2^) and 24 h incubation with a lipid extract of microalgae *Nannochloropsis oceanica* (algae, 3 ng/mL). Data obtained using high-performance liquid chromatography system Ultimate 3000 (Dionex, Idstein, Germany) coupled Q Exactive HF mass spectrometer (Thermo Fisher Scientific, Bremen, Germany). Raw data were analyzed by the MaxQuant v2.4.2 using the UniProtKB-SwissProt database (taxonomy: Homo sapiens, release 2023-09). Supplementary Table S3: Results of one-way univariate analysis of variance (ANOVA) of the proteins in keratinocytes isolated from the skin of healthy donors and psoriatic patients after UVB irradiation (60 mJ/cm^2^) and 24 h incubation with a lipid extract of the microalgae *Nannochloropsis oceanica* (3 ng/mL).
